# Supplementary material for: Resolving the full spectrum of human genome variation using Linked-Reads
Source: Genome Res. 2019 Apr;29(4):635–45. doi: 10.1101/gr.234443.118 (PMC6442396; doi:10.1101/gr.234443.118)
Supplement: Supplemental Material [file supp_gr.234443.118_Supplemental_longranger-2.2.zip › longranger-2.2.tar.bz2!/lr2.2-public/lib/go/src/code.google.com/vitess/docs/reference/vitess-api.html]

Vitess / Vitess API Reference


Toggle navigation

- Overview
- Guides
- Reference
- Blog
- GitHub

- #### Overview

  - What is Vitess
  - Scaling MySQL with Vitess
  - Key Concepts
- #### Getting Started

  - Run Vitess on Kubernetes
    - Custom Docker Build
  - Run Vitess Locally
- #### User Guide

  - Introduction- Client Libraries- Backing Up Data- Reparenting
        - Schema Management
        - VSchema Guide
        - Schema Swap (Tutorial)
        - Sharding
          - Horizontal Sharding (Tutorial, automated)
          - Horizontal Sharding (Tutorial, manual)
          - Sharding in Kubernetes (Tutorial, automated)
          - Sharding in Kubernetes (Tutorial, manual)
        - Vitess Sequences
        - MySQL Server Protocol
        - Vitess and Replication
        - Update Stream
        - Row Based Replication
        - Topology Service
        - Transport Security Model
        - Launching
          - Scalability Philosophy
          - Production Planning
          - Server Configuration
          - 2PC Guide
          - Troubleshooting
        - Upgrading
- #### Reference Guides

  - Vitess API- vtctl Commands
- #### Other Resources

  - Presentations- Blog- Roadmap
- #### Contributing

  - Contributing to Vitess- GitHub Workflow- Code Reviews
- GitHub

# Vitess API Reference

- #### Overview

  - What is Vitess
  - Scaling MySQL with Vitess
  - Key Concepts
- #### Getting Started

  - Run Vitess on Kubernetes
    - Custom Docker Build
  - Run Vitess Locally
- #### User Guide

  - Introduction- Client Libraries- Backing Up Data- Reparenting
        - Schema Management
        - VSchema Guide
        - Schema Swap (Tutorial)
        - Sharding
          - Horizontal Sharding (Tutorial, automated)
          - Horizontal Sharding (Tutorial, manual)
          - Sharding in Kubernetes (Tutorial, automated)
          - Sharding in Kubernetes (Tutorial, manual)
        - Vitess Sequences
        - MySQL Server Protocol
        - Vitess and Replication
        - Update Stream
        - Row Based Replication
        - Topology Service
        - Transport Security Model
        - Launching
          - Scalability Philosophy
          - Production Planning
          - Server Configuration
          - 2PC Guide
          - Troubleshooting
        - Upgrading
- #### Reference Guides

  - Vitess API- vtctl Commands
- #### Other Resources

  - Presentations- Blog- Roadmap
- #### Contributing

  - Contributing to Vitess- GitHub Workflow- Code Reviews

This document describes Vitess API methods that enable your client application to more easily talk to your storage system to query data. API methods are grouped into the following categories:

- Range-based Sharding
- Transactions
- Custom Sharding
- Map Reduce
- Topology
- v3 API (alpha)

The following table lists the methods in each group and links to more detail about each method:

|  |  |
| --- | --- |
| Range-based Sharding | |
| `ExecuteBatchKeyspaceIds` | ExecuteBatchKeyspaceIds executes the list of queries based on the specified keyspace ids. |
| `ExecuteEntityIds` | ExecuteEntityIds executes the query based on the specified external id to keyspace id map. |
| `ExecuteKeyRanges` | ExecuteKeyRanges executes the query based on the specified key ranges. |
| `ExecuteKeyspaceIds` | ExecuteKeyspaceIds executes the query based on the specified keyspace ids. |
| `StreamExecuteKeyRanges` | StreamExecuteKeyRanges executes a streaming query based on key ranges. Use this method if the query returns a large number of rows. |
| `StreamExecuteKeyspaceIds` | StreamExecuteKeyspaceIds executes a streaming query based on keyspace ids. Use this method if the query returns a large number of rows. |
| Transactions | |
| `Begin` | Begin a transaction. |
| `Commit` | Commit a transaction. |
| `ResolveTransaction` | ResolveTransaction resolves a transaction. |
| `Rollback` | Rollback a transaction. |
| Custom Sharding | |
| `ExecuteBatchShards` | ExecuteBatchShards executes the list of queries on the specified shards. |
| `ExecuteShards` | ExecuteShards executes the query on the specified shards. |
| `StreamExecuteShards` | StreamExecuteShards executes a streaming query based on shards. Use this method if the query returns a large number of rows. |
| Map Reduce | |
| `SplitQuery` | Split a query into non-overlapping sub queries |
| Topology | |
| `GetSrvKeyspace` | GetSrvKeyspace returns a SrvKeyspace object (as seen by this vtgate). This method is provided as a convenient way for clients to take a look at the sharding configuration for a Keyspace. Looking at the sharding information should not be used for routing queries (as the information may change, use the Execute calls for that). It is convenient for monitoring applications for instance, or if using custom sharding. |
| v3 API (alpha) | |
| `Execute` | Execute tries to route the query to the right shard. It depends on the query and bind variables to provide enough information in conjonction with the vindexes to route the query. |
| `StreamExecute` | StreamExecute executes a streaming query based on shards. It depends on the query and bind variables to provide enough information in conjonction with the vindexes to route the query. Use this method if the query returns a large number of rows. |

## Range-based Sharding

### ExecuteBatchKeyspaceIds

ExecuteBatchKeyspaceIds executes the list of queries based on the specified keyspace ids.

#### Request

ExecuteBatchKeyspaceIdsRequest is the payload to ExecuteBatchKeyspaceId.

##### Parameters

| Name | Description |
| --- | --- |
| `caller_id`  vtrpc.CallerID | CallerID is passed along RPCs to identify the originating client for a request. It is not meant to be secure, but only informational. The client can put whatever info they want in these fields, and they will be trusted by the servers. The fields will just be used for logging purposes, and to easily find a client. VtGate propagates it to VtTablet, and VtTablet may use this information for monitoring purposes, to display on dashboards, or for blacklisting purposes. |
| `session`  Session | Session objects are session cookies and are invalidated on use. Query results will contain updated session values. Their content should be opaque to the user. |
| `queries`  list <BoundKeyspaceIdQuery> | BoundKeyspaceIdQuery represents a single query request for the specified list of keyspace ids. This is used in a list for ExecuteBatchKeyspaceIdsRequest. |
| `tablet_type`  topodata.TabletType | TabletType represents the type of a given tablet. |
| `as_transaction`  bool | as\_transaction will execute the queries in this batch in a single transaction per shard, created for this purpose. (this can be seen as adding a 'begin' before and 'commit' after the queries). Only makes sense if tablet\_type is master. If set, the Session is ignored. |
| `options`  query.ExecuteOptions | ExecuteOptions is passed around for all Execute calls. |

#### Response

ExecuteBatchKeyspaceIdsResponse is the returned value from ExecuteBatchKeyspaceId.

##### Properties

| Name | Description |
| --- | --- |
| `error`  vtrpc.RPCError | RPCError is an application-level error structure returned by VtTablet (and passed along by VtGate if appropriate). We use this so the clients don't have to parse the error messages, but instead can depend on the value of the code. |
| `session`  Session | Session objects are session cookies and are invalidated on use. Query results will contain updated session values. Their content should be opaque to the user. |
| `results`  list <query.QueryResult> | QueryResult is returned by Execute and ExecuteStream. As returned by Execute, len(fields) is always equal to len(row) (for each row in rows). As returned by StreamExecute, the first QueryResult has the fields set, and subsequent QueryResult have rows set. And as Execute, len(QueryResult[0].fields) is always equal to len(row) (for each row in rows for each QueryResult in QueryResult[1:]). |

### ExecuteEntityIds

ExecuteEntityIds executes the query based on the specified external id to keyspace id map.

#### Request

ExecuteEntityIdsRequest is the payload to ExecuteEntityIds.

##### Parameters

| Name | Description |
| --- | --- |
| `caller_id`  vtrpc.CallerID | CallerID is passed along RPCs to identify the originating client for a request. It is not meant to be secure, but only informational. The client can put whatever info they want in these fields, and they will be trusted by the servers. The fields will just be used for logging purposes, and to easily find a client. VtGate propagates it to VtTablet, and VtTablet may use this information for monitoring purposes, to display on dashboards, or for blacklisting purposes. |
| `session`  Session | Session objects are session cookies and are invalidated on use. Query results will contain updated session values. Their content should be opaque to the user. |
| `query`  query.BoundQuery | BoundQuery is a query with its bind variables |
| `keyspace`  string | keyspace to target the query to. |
| `entity_column_name`  string | entity\_column\_name is the column name to use. |
| `entity_keyspace_ids`  list <EntityId> | entity\_keyspace\_ids are pairs of entity\_column\_name values associated with its corresponding keyspace\_id. |
| `tablet_type`  topodata.TabletType | TabletType represents the type of a given tablet. |
| `not_in_transaction`  bool | not\_in\_transaction is deprecated and should not be used. |
| `options`  query.ExecuteOptions | ExecuteOptions is passed around for all Execute calls. |

#### Messages

##### ExecuteEntityIdsRequest.EntityId

*Properties*

| Name | Description |
| --- | --- |
| `type`  query.Type | Type defines the various supported data types in bind vars and query results. |
| `value`  bytes | value is the value for the entity. Not set if type is NULL\_TYPE. |
| `keyspace_id`  bytes | keyspace\_id is the associated keyspace\_id for the entity. |

#### Response

ExecuteEntityIdsResponse is the returned value from ExecuteEntityIds.

##### Properties

| Name | Description |
| --- | --- |
| `error`  vtrpc.RPCError | RPCError is an application-level error structure returned by VtTablet (and passed along by VtGate if appropriate). We use this so the clients don't have to parse the error messages, but instead can depend on the value of the code. |
| `session`  Session | Session objects are session cookies and are invalidated on use. Query results will contain updated session values. Their content should be opaque to the user. |
| `result`  query.QueryResult | QueryResult is returned by Execute and ExecuteStream. As returned by Execute, len(fields) is always equal to len(row) (for each row in rows). As returned by StreamExecute, the first QueryResult has the fields set, and subsequent QueryResult have rows set. And as Execute, len(QueryResult[0].fields) is always equal to len(row) (for each row in rows for each QueryResult in QueryResult[1:]). |

### ExecuteKeyRanges

ExecuteKeyRanges executes the query based on the specified key ranges.

#### Request

ExecuteKeyRangesRequest is the payload to ExecuteKeyRanges.

##### Parameters

| Name | Description |
| --- | --- |
| `caller_id`  vtrpc.CallerID | CallerID is passed along RPCs to identify the originating client for a request. It is not meant to be secure, but only informational. The client can put whatever info they want in these fields, and they will be trusted by the servers. The fields will just be used for logging purposes, and to easily find a client. VtGate propagates it to VtTablet, and VtTablet may use this information for monitoring purposes, to display on dashboards, or for blacklisting purposes. |
| `session`  Session | Session objects are session cookies and are invalidated on use. Query results will contain updated session values. Their content should be opaque to the user. |
| `query`  query.BoundQuery | BoundQuery is a query with its bind variables |
| `keyspace`  string | keyspace to target the query to |
| `key_ranges`  list <topodata.KeyRange> | KeyRange describes a range of sharding keys, when range-based sharding is used. |
| `tablet_type`  topodata.TabletType | TabletType represents the type of a given tablet. |
| `not_in_transaction`  bool | not\_in\_transaction is deprecated and should not be used. |
| `options`  query.ExecuteOptions | ExecuteOptions is passed around for all Execute calls. |

#### Response

ExecuteKeyRangesResponse is the returned value from ExecuteKeyRanges.

##### Properties

| Name | Description |
| --- | --- |
| `error`  vtrpc.RPCError | RPCError is an application-level error structure returned by VtTablet (and passed along by VtGate if appropriate). We use this so the clients don't have to parse the error messages, but instead can depend on the value of the code. |
| `session`  Session | Session objects are session cookies and are invalidated on use. Query results will contain updated session values. Their content should be opaque to the user. |
| `result`  query.QueryResult | QueryResult is returned by Execute and ExecuteStream. As returned by Execute, len(fields) is always equal to len(row) (for each row in rows). As returned by StreamExecute, the first QueryResult has the fields set, and subsequent QueryResult have rows set. And as Execute, len(QueryResult[0].fields) is always equal to len(row) (for each row in rows for each QueryResult in QueryResult[1:]). |

### ExecuteKeyspaceIds

ExecuteKeyspaceIds executes the query based on the specified keyspace ids.

#### Request

ExecuteKeyspaceIdsRequest is the payload to ExecuteKeyspaceIds.

##### Parameters

| Name | Description |
| --- | --- |
| `caller_id`  vtrpc.CallerID | CallerID is passed along RPCs to identify the originating client for a request. It is not meant to be secure, but only informational. The client can put whatever info they want in these fields, and they will be trusted by the servers. The fields will just be used for logging purposes, and to easily find a client. VtGate propagates it to VtTablet, and VtTablet may use this information for monitoring purposes, to display on dashboards, or for blacklisting purposes. |
| `session`  Session | Session objects are session cookies and are invalidated on use. Query results will contain updated session values. Their content should be opaque to the user. |
| `query`  query.BoundQuery | BoundQuery is a query with its bind variables |
| `keyspace`  string | keyspace to target the query to. |
| `keyspace_ids`  list <bytes> | keyspace\_ids contains the list of keyspace\_ids affected by this query. Will be used to find the shards to send the query to. |
| `tablet_type`  topodata.TabletType | TabletType represents the type of a given tablet. |
| `not_in_transaction`  bool | not\_in\_transaction is deprecated and should not be used. |
| `options`  query.ExecuteOptions | ExecuteOptions is passed around for all Execute calls. |

#### Response

ExecuteKeyspaceIdsResponse is the returned value from ExecuteKeyspaceIds.

##### Properties

| Name | Description |
| --- | --- |
| `error`  vtrpc.RPCError | RPCError is an application-level error structure returned by VtTablet (and passed along by VtGate if appropriate). We use this so the clients don't have to parse the error messages, but instead can depend on the value of the code. |
| `session`  Session | Session objects are session cookies and are invalidated on use. Query results will contain updated session values. Their content should be opaque to the user. |
| `result`  query.QueryResult | QueryResult is returned by Execute and ExecuteStream. As returned by Execute, len(fields) is always equal to len(row) (for each row in rows). As returned by StreamExecute, the first QueryResult has the fields set, and subsequent QueryResult have rows set. And as Execute, len(QueryResult[0].fields) is always equal to len(row) (for each row in rows for each QueryResult in QueryResult[1:]). |

### StreamExecuteKeyRanges

StreamExecuteKeyRanges executes a streaming query based on key ranges. Use this method if the query returns a large number of rows.

#### Request

StreamExecuteKeyRangesRequest is the payload to StreamExecuteKeyRanges.

##### Parameters

| Name | Description |
| --- | --- |
| `caller_id`  vtrpc.CallerID | CallerID is passed along RPCs to identify the originating client for a request. It is not meant to be secure, but only informational. The client can put whatever info they want in these fields, and they will be trusted by the servers. The fields will just be used for logging purposes, and to easily find a client. VtGate propagates it to VtTablet, and VtTablet may use this information for monitoring purposes, to display on dashboards, or for blacklisting purposes. |
| `query`  query.BoundQuery | BoundQuery is a query with its bind variables |
| `keyspace`  string | keyspace to target the query to. |
| `key_ranges`  list <topodata.KeyRange> | KeyRange describes a range of sharding keys, when range-based sharding is used. |
| `tablet_type`  topodata.TabletType | TabletType represents the type of a given tablet. |
| `options`  query.ExecuteOptions | ExecuteOptions is passed around for all Execute calls. |

#### Response

StreamExecuteKeyRangesResponse is the returned value from StreamExecuteKeyRanges.

##### Properties

| Name | Description |
| --- | --- |
| `result`  query.QueryResult | QueryResult is returned by Execute and ExecuteStream. As returned by Execute, len(fields) is always equal to len(row) (for each row in rows). As returned by StreamExecute, the first QueryResult has the fields set, and subsequent QueryResult have rows set. And as Execute, len(QueryResult[0].fields) is always equal to len(row) (for each row in rows for each QueryResult in QueryResult[1:]). |

### StreamExecuteKeyspaceIds

StreamExecuteKeyspaceIds executes a streaming query based on keyspace ids. Use this method if the query returns a large number of rows.

#### Request

StreamExecuteKeyspaceIdsRequest is the payload to StreamExecuteKeyspaceIds.

##### Parameters

| Name | Description |
| --- | --- |
| `caller_id`  vtrpc.CallerID | CallerID is passed along RPCs to identify the originating client for a request. It is not meant to be secure, but only informational. The client can put whatever info they want in these fields, and they will be trusted by the servers. The fields will just be used for logging purposes, and to easily find a client. VtGate propagates it to VtTablet, and VtTablet may use this information for monitoring purposes, to display on dashboards, or for blacklisting purposes. |
| `query`  query.BoundQuery | BoundQuery is a query with its bind variables |
| `keyspace`  string | keyspace to target the query to. |
| `keyspace_ids`  list <bytes> | keyspace\_ids contains the list of keyspace\_ids affected by this query. Will be used to find the shards to send the query to. |
| `tablet_type`  topodata.TabletType | TabletType represents the type of a given tablet. |
| `options`  query.ExecuteOptions | ExecuteOptions is passed around for all Execute calls. |

#### Response

StreamExecuteKeyspaceIdsResponse is the returned value from StreamExecuteKeyspaceIds.

##### Properties

| Name | Description |
| --- | --- |
| `result`  query.QueryResult | QueryResult is returned by Execute and ExecuteStream. As returned by Execute, len(fields) is always equal to len(row) (for each row in rows). As returned by StreamExecute, the first QueryResult has the fields set, and subsequent QueryResult have rows set. And as Execute, len(QueryResult[0].fields) is always equal to len(row) (for each row in rows for each QueryResult in QueryResult[1:]). |

## Transactions

### Begin

Begin a transaction.

#### Request

BeginRequest is the payload to Begin.

##### Parameters

| Name | Description |
| --- | --- |
| `caller_id`  vtrpc.CallerID | CallerID is passed along RPCs to identify the originating client for a request. It is not meant to be secure, but only informational. The client can put whatever info they want in these fields, and they will be trusted by the servers. The fields will just be used for logging purposes, and to easily find a client. VtGate propagates it to VtTablet, and VtTablet may use this information for monitoring purposes, to display on dashboards, or for blacklisting purposes. |
| `single_db`  bool | single\_db specifies if the transaction should be restricted to a single database. |

#### Response

BeginResponse is the returned value from Begin.

##### Properties

| Name | Description |
| --- | --- |
| `session`  Session | Session objects are session cookies and are invalidated on use. Query results will contain updated session values. Their content should be opaque to the user. |

### Commit

Commit a transaction.

#### Request

CommitRequest is the payload to Commit.

##### Parameters

| Name | Description |
| --- | --- |
| `caller_id`  vtrpc.CallerID | CallerID is passed along RPCs to identify the originating client for a request. It is not meant to be secure, but only informational. The client can put whatever info they want in these fields, and they will be trusted by the servers. The fields will just be used for logging purposes, and to easily find a client. VtGate propagates it to VtTablet, and VtTablet may use this information for monitoring purposes, to display on dashboards, or for blacklisting purposes. |
| `session`  Session | Session objects are session cookies and are invalidated on use. Query results will contain updated session values. Their content should be opaque to the user. |
| `atomic`  bool | atomic specifies if the commit should go through the 2PC workflow to ensure atomicity. |

#### Response

CommitResponse is the returned value from Commit.

##### Properties

| Name | Description |
| --- | --- |

### ResolveTransaction

ResolveTransaction resolves a transaction.

#### Request

ResolveTransactionRequest is the payload to ResolveTransaction.

##### Parameters

| Name | Description |
| --- | --- |
| `caller_id`  vtrpc.CallerID | CallerID is passed along RPCs to identify the originating client for a request. It is not meant to be secure, but only informational. The client can put whatever info they want in these fields, and they will be trusted by the servers. The fields will just be used for logging purposes, and to easily find a client. VtGate propagates it to VtTablet, and VtTablet may use this information for monitoring purposes, to display on dashboards, or for blacklisting purposes. |
| `dtid`  string | dtid is the dtid of the transaction to be resolved. |

#### Response

ResolveTransactionResponse is the returned value from Rollback.

##### Properties

| Name | Description |
| --- | --- |

### Rollback

Rollback a transaction.

#### Request

RollbackRequest is the payload to Rollback.

##### Parameters

| Name | Description |
| --- | --- |
| `caller_id`  vtrpc.CallerID | CallerID is passed along RPCs to identify the originating client for a request. It is not meant to be secure, but only informational. The client can put whatever info they want in these fields, and they will be trusted by the servers. The fields will just be used for logging purposes, and to easily find a client. VtGate propagates it to VtTablet, and VtTablet may use this information for monitoring purposes, to display on dashboards, or for blacklisting purposes. |
| `session`  Session | Session objects are session cookies and are invalidated on use. Query results will contain updated session values. Their content should be opaque to the user. |

#### Response

RollbackResponse is the returned value from Rollback.

##### Properties

| Name | Description |
| --- | --- |

## Custom Sharding

### ExecuteBatchShards

ExecuteBatchShards executes the list of queries on the specified shards.

#### Request

ExecuteBatchShardsRequest is the payload to ExecuteBatchShards

##### Parameters

| Name | Description |
| --- | --- |
| `caller_id`  vtrpc.CallerID | CallerID is passed along RPCs to identify the originating client for a request. It is not meant to be secure, but only informational. The client can put whatever info they want in these fields, and they will be trusted by the servers. The fields will just be used for logging purposes, and to easily find a client. VtGate propagates it to VtTablet, and VtTablet may use this information for monitoring purposes, to display on dashboards, or for blacklisting purposes. |
| `session`  Session | Session objects are session cookies and are invalidated on use. Query results will contain updated session values. Their content should be opaque to the user. |
| `queries`  list <BoundShardQuery> | BoundShardQuery represents a single query request for the specified list of shards. This is used in a list for ExecuteBatchShardsRequest. |
| `tablet_type`  topodata.TabletType | TabletType represents the type of a given tablet. |
| `as_transaction`  bool | as\_transaction will execute the queries in this batch in a single transaction per shard, created for this purpose. (this can be seen as adding a 'begin' before and 'commit' after the queries). Only makes sense if tablet\_type is master. If set, the Session is ignored. |
| `options`  query.ExecuteOptions | ExecuteOptions is passed around for all Execute calls. |

#### Response

ExecuteBatchShardsResponse is the returned value from ExecuteBatchShards.

##### Properties

| Name | Description |
| --- | --- |
| `error`  vtrpc.RPCError | RPCError is an application-level error structure returned by VtTablet (and passed along by VtGate if appropriate). We use this so the clients don't have to parse the error messages, but instead can depend on the value of the code. |
| `session`  Session | Session objects are session cookies and are invalidated on use. Query results will contain updated session values. Their content should be opaque to the user. |
| `results`  list <query.QueryResult> | QueryResult is returned by Execute and ExecuteStream. As returned by Execute, len(fields) is always equal to len(row) (for each row in rows). As returned by StreamExecute, the first QueryResult has the fields set, and subsequent QueryResult have rows set. And as Execute, len(QueryResult[0].fields) is always equal to len(row) (for each row in rows for each QueryResult in QueryResult[1:]). |

### ExecuteShards

ExecuteShards executes the query on the specified shards.

#### Request

ExecuteShardsRequest is the payload to ExecuteShards.

##### Parameters

| Name | Description |
| --- | --- |
| `caller_id`  vtrpc.CallerID | CallerID is passed along RPCs to identify the originating client for a request. It is not meant to be secure, but only informational. The client can put whatever info they want in these fields, and they will be trusted by the servers. The fields will just be used for logging purposes, and to easily find a client. VtGate propagates it to VtTablet, and VtTablet may use this information for monitoring purposes, to display on dashboards, or for blacklisting purposes. |
| `session`  Session | Session objects are session cookies and are invalidated on use. Query results will contain updated session values. Their content should be opaque to the user. |
| `query`  query.BoundQuery | BoundQuery is a query with its bind variables |
| `keyspace`  string | keyspace to target the query to. |
| `shards`  list <string> | shards to target the query to. A DML can only target one shard. |
| `tablet_type`  topodata.TabletType | TabletType represents the type of a given tablet. |
| `not_in_transaction`  bool | not\_in\_transaction is deprecated and should not be used. |
| `options`  query.ExecuteOptions | ExecuteOptions is passed around for all Execute calls. |

#### Response

ExecuteShardsResponse is the returned value from ExecuteShards.

##### Properties

| Name | Description |
| --- | --- |
| `error`  vtrpc.RPCError | RPCError is an application-level error structure returned by VtTablet (and passed along by VtGate if appropriate). We use this so the clients don't have to parse the error messages, but instead can depend on the value of the code. |
| `session`  Session | Session objects are session cookies and are invalidated on use. Query results will contain updated session values. Their content should be opaque to the user. |
| `result`  query.QueryResult | QueryResult is returned by Execute and ExecuteStream. As returned by Execute, len(fields) is always equal to len(row) (for each row in rows). As returned by StreamExecute, the first QueryResult has the fields set, and subsequent QueryResult have rows set. And as Execute, len(QueryResult[0].fields) is always equal to len(row) (for each row in rows for each QueryResult in QueryResult[1:]). |

### StreamExecuteShards

StreamExecuteShards executes a streaming query based on shards. Use this method if the query returns a large number of rows.

#### Request

StreamExecuteShardsRequest is the payload to StreamExecuteShards.

##### Parameters

| Name | Description |
| --- | --- |
| `caller_id`  vtrpc.CallerID | CallerID is passed along RPCs to identify the originating client for a request. It is not meant to be secure, but only informational. The client can put whatever info they want in these fields, and they will be trusted by the servers. The fields will just be used for logging purposes, and to easily find a client. VtGate propagates it to VtTablet, and VtTablet may use this information for monitoring purposes, to display on dashboards, or for blacklisting purposes. |
| `query`  query.BoundQuery | BoundQuery is a query with its bind variables |
| `keyspace`  string | keyspace to target the query to. |
| `shards`  list <string> | shards to target the query to. |
| `tablet_type`  topodata.TabletType | TabletType represents the type of a given tablet. |
| `options`  query.ExecuteOptions | ExecuteOptions is passed around for all Execute calls. |

#### Response

StreamExecuteShardsResponse is the returned value from StreamExecuteShards.

##### Properties

| Name | Description |
| --- | --- |
| `result`  query.QueryResult | QueryResult is returned by Execute and ExecuteStream. As returned by Execute, len(fields) is always equal to len(row) (for each row in rows). As returned by StreamExecute, the first QueryResult has the fields set, and subsequent QueryResult have rows set. And as Execute, len(QueryResult[0].fields) is always equal to len(row) (for each row in rows for each QueryResult in QueryResult[1:]). |

## Map Reduce

### SplitQuery

Split a query into non-overlapping sub queries

#### Request

SplitQueryRequest is the payload to SplitQuery. SplitQuery takes a "SELECT" query and generates a list of queries called "query-parts". Each query-part consists of the original query with an added WHERE clause that restricts the query-part to operate only on rows whose values in the the columns listed in the "split\_column" field of the request (see below) are in a particular range. It is guaranteed that the set of rows obtained from executing each query-part on a database snapshot and merging (without deduping) the results is equal to the set of rows obtained from executing the original query on the same snapshot with the rows containing NULL values in any of the split\_column's excluded. This is typically called by the MapReduce master when reading from Vitess. There it's desirable that the sets of rows returned by the query-parts have roughly the same size.

##### Parameters

| Name | Description |
| --- | --- |
| `caller_id`  vtrpc.CallerID | CallerID is passed along RPCs to identify the originating client for a request. It is not meant to be secure, but only informational. The client can put whatever info they want in these fields, and they will be trusted by the servers. The fields will just be used for logging purposes, and to easily find a client. VtGate propagates it to VtTablet, and VtTablet may use this information for monitoring purposes, to display on dashboards, or for blacklisting purposes. |
| `keyspace`  string | keyspace to target the query to. |
| `query`  query.BoundQuery | BoundQuery is a query with its bind variables |
| `split_column`  list <string> | Each generated query-part will be restricted to rows whose values in the columns listed in this field are in a particular range. The list of columns named here must be a prefix of the list of columns defining some index or primary key of the table referenced in 'query'. For many tables using the primary key columns (in order) is sufficient and this is the default if this field is omitted. See the comment on the 'algorithm' field for more restrictions and information. |
| `split_count`  int64 | You can specify either an estimate of the number of query-parts to generate or an estimate of the number of rows each query-part should return. Thus, exactly one of split\_count or num\_rows\_per\_query\_part should be nonzero. The non-given parameter is calculated from the given parameter using the formula: split\_count \* num\_rows\_per\_query\_pary = table\_size, where table\_size is an approximation of the number of rows in the table. Note that if "split\_count" is given it is regarded as an estimate. The number of query-parts returned may differ slightly (in particular, if it's not a whole multiple of the number of vitess shards). |
| `num_rows_per_query_part`  int64 |  |
| `algorithm`  query.SplitQueryRequest.Algorithm | The algorithm to use to split the query. The split algorithm is performed on each database shard in parallel. The lists of query-parts generated by the shards are merged and returned to the caller. Two algorithms are supported: EQUAL\_SPLITS If this algorithm is selected then only the first 'split\_column' given is used (or the first primary key column if the 'split\_column' field is empty). In the rest of this algorithm's description, we refer to this column as "the split column". The split column must have numeric type (integral or floating point). The algorithm works by taking the interval [min, max], where min and max are the minimum and maximum values of the split column in the table-shard, respectively, and partitioning it into 'split\_count' sub-intervals of equal size. The added WHERE clause of each query-part restricts that part to rows whose value in the split column belongs to a particular sub-interval. This is fast, but requires that the distribution of values of the split column be uniform in [min, max] for the number of rows returned by each query part to be roughly the same. FULL\_SCAN If this algorithm is used then the split\_column must be the primary key columns (in order). This algorithm performs a full-scan of the table-shard referenced in 'query' to get "boundary" rows that are num\_rows\_per\_query\_part apart when the table is ordered by the columns listed in 'split\_column'. It then restricts each query-part to the rows located between two successive boundary rows. This algorithm supports multiple split\_column's of any type, but is slower than EQUAL\_SPLITS. |
| `use_split_query_v2`  bool | Remove this field after this new server code is released to prod. We must keep it for now, so that clients can still send it to the old server code currently in production. |

#### Response

SplitQueryResponse is the returned value from SplitQuery.

##### Properties

| Name | Description |
| --- | --- |
| `splits`  list <Part> | splits contains the queries to run to fetch the entire data set. |

#### Messages

##### SplitQueryResponse.KeyRangePart

*Properties*

| Name | Description |
| --- | --- |
| `keyspace`  string | keyspace to target the query to. |
| `key_ranges`  list <topodata.KeyRange> | KeyRange describes a range of sharding keys, when range-based sharding is used. |

##### SplitQueryResponse.Part

*Properties*

| Name | Description |
| --- | --- |
| `query`  query.BoundQuery | BoundQuery is a query with its bind variables |
| `key_range_part`  KeyRangePart | key\_range\_part is set if the query should be executed by ExecuteKeyRanges. |
| `shard_part`  ShardPart | shard\_part is set if the query should be executed by ExecuteShards. |
| `size`  int64 | size is the approximate number of rows this query will return. |

##### SplitQueryResponse.ShardPart

*Properties*

| Name | Description |
| --- | --- |
| `keyspace`  string | keyspace to target the query to. |
| `shards`  list <string> | shards to target the query to. |

## Topology

### GetSrvKeyspace

GetSrvKeyspace returns a SrvKeyspace object (as seen by this vtgate). This method is provided as a convenient way for clients to take a look at the sharding configuration for a Keyspace. Looking at the sharding information should not be used for routing queries (as the information may change, use the Execute calls for that). It is convenient for monitoring applications for instance, or if using custom sharding.

#### Request

GetSrvKeyspaceRequest is the payload to GetSrvKeyspace.

##### Parameters

| Name | Description |
| --- | --- |
| `keyspace`  string | keyspace name to fetch. |

#### Response

GetSrvKeyspaceResponse is the returned value from GetSrvKeyspace.

##### Properties

| Name | Description |
| --- | --- |
| `srv_keyspace`  topodata.SrvKeyspace | SrvKeyspace is a rollup node for the keyspace itself. |

## v3 API (alpha)

### Execute

Execute tries to route the query to the right shard. It depends on the query and bind variables to provide enough information in conjonction with the vindexes to route the query.

#### Request

ExecuteRequest is the payload to Execute.

##### Parameters

| Name | Description |
| --- | --- |
| `caller_id`  vtrpc.CallerID | CallerID is passed along RPCs to identify the originating client for a request. It is not meant to be secure, but only informational. The client can put whatever info they want in these fields, and they will be trusted by the servers. The fields will just be used for logging purposes, and to easily find a client. VtGate propagates it to VtTablet, and VtTablet may use this information for monitoring purposes, to display on dashboards, or for blacklisting purposes. |
| `session`  Session | Session objects are session cookies and are invalidated on use. Query results will contain updated session values. Their content should be opaque to the user. |
| `query`  query.BoundQuery | BoundQuery is a query with its bind variables |
| `tablet_type`  topodata.TabletType | TabletType represents the type of a given tablet. |
| `not_in_transaction`  bool | not\_in\_transaction is deprecated and should not be used. |
| `keyspace`  string | keyspace to target the query to. |
| `options`  query.ExecuteOptions | ExecuteOptions is passed around for all Execute calls. |

#### Response

ExecuteResponse is the returned value from Execute.

##### Properties

| Name | Description |
| --- | --- |
| `error`  vtrpc.RPCError | RPCError is an application-level error structure returned by VtTablet (and passed along by VtGate if appropriate). We use this so the clients don't have to parse the error messages, but instead can depend on the value of the code. |
| `session`  Session | Session objects are session cookies and are invalidated on use. Query results will contain updated session values. Their content should be opaque to the user. |
| `result`  query.QueryResult | QueryResult is returned by Execute and ExecuteStream. As returned by Execute, len(fields) is always equal to len(row) (for each row in rows). As returned by StreamExecute, the first QueryResult has the fields set, and subsequent QueryResult have rows set. And as Execute, len(QueryResult[0].fields) is always equal to len(row) (for each row in rows for each QueryResult in QueryResult[1:]). |

### StreamExecute

StreamExecute executes a streaming query based on shards. It depends on the query and bind variables to provide enough information in conjonction with the vindexes to route the query. Use this method if the query returns a large number of rows.

#### Request

StreamExecuteRequest is the payload to StreamExecute.

##### Parameters

| Name | Description |
| --- | --- |
| `caller_id`  vtrpc.CallerID | CallerID is passed along RPCs to identify the originating client for a request. It is not meant to be secure, but only informational. The client can put whatever info they want in these fields, and they will be trusted by the servers. The fields will just be used for logging purposes, and to easily find a client. VtGate propagates it to VtTablet, and VtTablet may use this information for monitoring purposes, to display on dashboards, or for blacklisting purposes. |
| `query`  query.BoundQuery | BoundQuery is a query with its bind variables |
| `tablet_type`  topodata.TabletType | TabletType represents the type of a given tablet. |
| `keyspace`  string | keyspace to target the query to. |
| `options`  query.ExecuteOptions | ExecuteOptions is passed around for all Execute calls. |

#### Response

StreamExecuteResponse is the returned value from StreamExecute.

##### Properties

| Name | Description |
| --- | --- |
| `result`  query.QueryResult | QueryResult is returned by Execute and ExecuteStream. As returned by Execute, len(fields) is always equal to len(row) (for each row in rows). As returned by StreamExecute, the first QueryResult has the fields set, and subsequent QueryResult have rows set. And as Execute, len(QueryResult[0].fields) is always equal to len(row) (for each row in rows for each QueryResult in QueryResult[1:]). |

## Enums

### query.Type

Type defines the various supported data types in bind vars and query results.

| Name | Value | Description |
| --- | --- | --- |
| `NULL_TYPE` | `0` | NULL\_TYPE specifies a NULL type. |
| `INT8` | `257` | INT8 specifies a TINYINT type. Properties: 1, IsNumber. |
| `UINT8` | `770` | UINT8 specifies a TINYINT UNSIGNED type. Properties: 2, IsNumber, IsUnsigned. |
| `INT16` | `259` | INT16 specifies a SMALLINT type. Properties: 3, IsNumber. |
| `UINT16` | `772` | UINT16 specifies a SMALLINT UNSIGNED type. Properties: 4, IsNumber, IsUnsigned. |
| `INT24` | `261` | INT24 specifies a MEDIUMINT type. Properties: 5, IsNumber. |
| `UINT24` | `774` | UINT24 specifies a MEDIUMINT UNSIGNED type. Properties: 6, IsNumber, IsUnsigned. |
| `INT32` | `263` | INT32 specifies a INTEGER type. Properties: 7, IsNumber. |
| `UINT32` | `776` | UINT32 specifies a INTEGER UNSIGNED type. Properties: 8, IsNumber, IsUnsigned. |
| `INT64` | `265` | INT64 specifies a BIGINT type. Properties: 9, IsNumber. |
| `UINT64` | `778` | UINT64 specifies a BIGINT UNSIGNED type. Properties: 10, IsNumber, IsUnsigned. |
| `FLOAT32` | `1035` | FLOAT32 specifies a FLOAT type. Properties: 11, IsFloat. |
| `FLOAT64` | `1036` | FLOAT64 specifies a DOUBLE or REAL type. Properties: 12, IsFloat. |
| `TIMESTAMP` | `2061` | TIMESTAMP specifies a TIMESTAMP type. Properties: 13, IsQuoted. |
| `DATE` | `2062` | DATE specifies a DATE type. Properties: 14, IsQuoted. |
| `TIME` | `2063` | TIME specifies a TIME type. Properties: 15, IsQuoted. |
| `DATETIME` | `2064` | DATETIME specifies a DATETIME type. Properties: 16, IsQuoted. |
| `YEAR` | `785` | YEAR specifies a YEAR type. Properties: 17, IsNumber, IsUnsigned. |
| `DECIMAL` | `18` | DECIMAL specifies a DECIMAL or NUMERIC type. Properties: 18, None. |
| `TEXT` | `6163` | TEXT specifies a TEXT type. Properties: 19, IsQuoted, IsText. |
| `BLOB` | `10260` | BLOB specifies a BLOB type. Properties: 20, IsQuoted, IsBinary. |
| `VARCHAR` | `6165` | VARCHAR specifies a VARCHAR type. Properties: 21, IsQuoted, IsText. |
| `VARBINARY` | `10262` | VARBINARY specifies a VARBINARY type. Properties: 22, IsQuoted, IsBinary. |
| `CHAR` | `6167` | CHAR specifies a CHAR type. Properties: 23, IsQuoted, IsText. |
| `BINARY` | `10264` | BINARY specifies a BINARY type. Properties: 24, IsQuoted, IsBinary. |
| `BIT` | `2073` | BIT specifies a BIT type. Properties: 25, IsQuoted. |
| `ENUM` | `2074` | ENUM specifies an ENUM type. Properties: 26, IsQuoted. |
| `SET` | `2075` | SET specifies a SET type. Properties: 27, IsQuoted. |
| `TUPLE` | `28` | TUPLE specifies a a tuple. This cannot be returned in a QueryResult, but it can be sent as a bind var. Properties: 28, None. |
| `GEOMETRY` | `2077` | GEOMETRY specifies a GEOMETRY type. Properties: 29, IsQuoted. |
| `JSON` | `2078` | JSON specified a JSON type. Properties: 30, IsQuoted. |

### topodata.KeyspaceIdType

KeyspaceIdType describes the type of the sharding key for a range-based sharded keyspace.

| Name | Value | Description |
| --- | --- | --- |
| `UNSET` | `0` | UNSET is the default value, when range-based sharding is not used. |
| `UINT64` | `1` | UINT64 is when uint64 value is used. This is represented as 'unsigned bigint' in mysql |
| `BYTES` | `2` | BYTES is when an array of bytes is used. This is represented as 'varbinary' in mysql |

### topodata.TabletType

TabletType represents the type of a given tablet.

| Name | Value | Description |
| --- | --- | --- |
| `UNKNOWN` | `0` | UNKNOWN is not a valid value. |
| `MASTER` | `1` | MASTER is the master server for the shard. Only MASTER allows DMLs. |
| `REPLICA` | `2` | REPLICA is a slave type. It is used to serve live traffic. A REPLICA can be promoted to MASTER. A demoted MASTER will go to REPLICA. |
| `RDONLY` | `3` | RDONLY (old name) / BATCH (new name) is used to serve traffic for long-running jobs. It is a separate type from REPLICA so long-running queries don't affect web-like traffic. |
| `BATCH` | `3` |  |
| `SPARE` | `4` | SPARE is a type of servers that cannot serve queries, but is available in case an extra server is needed. |
| `EXPERIMENTAL` | `5` | EXPERIMENTAL is like SPARE, except it can serve queries. This type can be used for usages not planned by Vitess, like online export to another storage engine. |
| `BACKUP` | `6` | BACKUP is the type a server goes to when taking a backup. No queries can be served in BACKUP mode. |
| `RESTORE` | `7` | RESTORE is the type a server uses when restoring a backup, at startup time. No queries can be served in RESTORE mode. |
| `DRAINED` | `8` | DRAINED is the type a server goes into when used by Vitess tools to perform an offline action. It is a serving type (as the tools processes may need to run queries), but it's not used to route queries from Vitess users. In this state, this tablet is dedicated to the process that uses it. |

### vtrpc.ErrorCode

ErrorCode is the enum values for Errors. Internally, errors should be created with one of these codes. These will then be translated over the wire by various RPC frameworks.

| Name | Value | Description |
| --- | --- | --- |
| `SUCCESS` | `0` | SUCCESS is returned from a successful call. |
| `CANCELLED` | `1` | CANCELLED means that the context was cancelled (and noticed in the app layer, as opposed to the RPC layer). |
| `UNKNOWN_ERROR` | `2` | UNKNOWN\_ERROR includes: 1. MySQL error codes that we don't explicitly handle. 2. MySQL response that wasn't as expected. For example, we might expect a MySQL timestamp to be returned in a particular way, but it wasn't. 3. Anything else that doesn't fall into a different bucket. |
| `BAD_INPUT` | `3` | BAD\_INPUT is returned when an end-user either sends SQL that couldn't be parsed correctly, or tries a query that isn't supported by Vitess. |
| `DEADLINE_EXCEEDED` | `4` | DEADLINE\_EXCEEDED is returned when an action is taking longer than a given timeout. |
| `INTEGRITY_ERROR` | `5` | INTEGRITY\_ERROR is returned on integrity error from MySQL, usually due to duplicate primary keys. |
| `PERMISSION_DENIED` | `6` | PERMISSION\_DENIED errors are returned when a user requests access to something that they don't have permissions for. |
| `RESOURCE_EXHAUSTED` | `7` | RESOURCE\_EXHAUSTED is returned when a query exceeds its quota in some dimension and can't be completed due to that. Queries that return RESOURCE\_EXHAUSTED should not be retried, as it could be detrimental to the server's health. Examples of errors that will cause the RESOURCE\_EXHAUSTED code: 1. TxPoolFull: this is retried server-side, and is only returned as an error if the server-side retries failed. 2. Query is killed due to it taking too long. |
| `QUERY_NOT_SERVED` | `8` | QUERY\_NOT\_SERVED means that a query could not be served right now. Client can interpret it as: "the tablet that you sent this query to cannot serve the query right now, try a different tablet or try again later." This could be due to various reasons: QueryService is not serving, should not be serving, wrong shard, wrong tablet type, blacklisted table, etc. Clients that receive this error should usually retry the query, but after taking the appropriate steps to make sure that the query will get sent to the correct tablet. |
| `NOT_IN_TX` | `9` | NOT\_IN\_TX means that we're not currently in a transaction, but we should be. |
| `INTERNAL_ERROR` | `10` | INTERNAL\_ERRORs are problems that only the server can fix, not the client. These errors are not due to a query itself, but rather due to the state of the system. Generally, we don't expect the errors to go away by themselves, but they may go away after human intervention. Examples of scenarios where INTERNAL\_ERROR is returned: 1. Something is not configured correctly internally. 2. A necessary resource is not available, and we don't expect it to become available by itself. 3. A sanity check fails. 4. Some other internal error occurs. Clients should not retry immediately, as there is little chance of success. However, it's acceptable for retries to happen internally, for example to multiple backends, in case only a subset of backend are not functional. |
| `TRANSIENT_ERROR` | `11` | TRANSIENT\_ERROR is used for when there is some error that we expect we can recover from automatically - often due to a resource limit temporarily being reached. Retrying this error, with an exponential backoff, should succeed. Clients should be able to successfully retry the query on the same backends. Examples of things that can trigger this error: 1. Query has been throttled 2. VtGate could have request backlog |
| `UNAUTHENTICATED` | `12` | UNAUTHENTICATED errors are returned when a user requests access to something, and we're unable to verify the user's authentication. |

## Messages

### BoundKeyspaceIdQuery

BoundKeyspaceIdQuery represents a single query request for the specified list of keyspace ids. This is used in a list for ExecuteBatchKeyspaceIdsRequest.

#### Properties

| Name | Description |
| --- | --- |
| `query`  query.BoundQuery | BoundQuery is a query with its bind variables |
| `keyspace`  string | keyspace to target the query to. |
| `keyspace_ids`  list <bytes> | keyspace\_ids contains the list of keyspace\_ids affected by this query. Will be used to find the shards to send the query to. |

### BoundShardQuery

BoundShardQuery represents a single query request for the specified list of shards. This is used in a list for ExecuteBatchShardsRequest.

#### Properties

| Name | Description |
| --- | --- |
| `query`  query.BoundQuery | BoundQuery is a query with its bind variables |
| `keyspace`  string | keyspace to target the query to. |
| `shards`  list <string> | shards to target the query to. A DML can only target one shard. |

### Session

Session objects are session cookies and are invalidated on use. Query results will contain updated session values. Their content should be opaque to the user.

#### Properties

| Name | Description |
| --- | --- |
| `in_transaction`  bool |  |
| `shard_sessions`  list <ShardSession> |  |
| `single_db`  bool | single\_db specifies if the transaction should be restricted to a single database. |

#### Messages

##### Session.ShardSession

*Properties*

| Name | Description |
| --- | --- |
| `target`  query.Target | Target describes what the client expects the tablet is. If the tablet does not match, an error is returned. |
| `transaction_id`  int64 |  |

### query.BindVariable

BindVariable represents a single bind variable in a Query.

#### Properties

| Name | Description |
| --- | --- |
| `type`  Type |  |
| `value`  bytes |  |
| `values`  list <Value> | Value represents a typed value. |

### query.BoundQuery

BoundQuery is a query with its bind variables

#### Properties

| Name | Description |
| --- | --- |
| `sql`  string | sql is the SQL query to execute |
| `bind_variables`  map <string, BindVariable> | bind\_variables is a map of all bind variables to expand in the query |

### query.EventToken

EventToken is a structure that describes a point in time in a replication stream on one shard. The most recent known replication position can be retrieved from vttablet when executing a query. It is also sent with the replication streams from the binlog service.

#### Properties

| Name | Description |
| --- | --- |
| `timestamp`  int64 | timestamp is the MySQL timestamp of the statements. Seconds since Epoch. |
| `shard`  string | The shard name that applied the statements. Note this is not set when streaming from a vttablet. It is only used on the client -> vtgate link. |
| `position`  string | The position on the replication stream after this statement was applied. It is not the transaction ID / GTID, but the position / GTIDSet. |

### query.ExecuteOptions

ExecuteOptions is passed around for all Execute calls.

#### Properties

| Name | Description |
| --- | --- |
| `include_event_token`  bool | This used to be exclude\_field\_names, which was replaced by IncludedFields enum below If set, we will try to include an EventToken with the responses. |
| `compare_event_token`  EventToken | EventToken is a structure that describes a point in time in a replication stream on one shard. The most recent known replication position can be retrieved from vttablet when executing a query. It is also sent with the replication streams from the binlog service. |
| `included_fields`  IncludedFields | Controls what fields are returned in Field message responses from mysql, i.e. field name, table name, etc. This is an optimization for high-QPS queries where the client knows what it's getting |

#### Enums

##### ExecuteOptions.IncludedFields

| Name | Value | Description |
| --- | --- | --- |
| `TYPE_AND_NAME` | `0` |  |
| `TYPE_ONLY` | `1` |  |
| `ALL` | `2` |  |

### query.Field

Field describes a single column returned by a query

#### Properties

| Name | Description |
| --- | --- |
| `name`  string | name of the field as returned by mysql C API |
| `type`  Type | vitess-defined type. Conversion function is in sqltypes package. |
| `table`  string | Remaining fields from mysql C API. These fields are only populated when ExecuteOptions.included\_fields is set to IncludedFields.ALL. |
| `org_table`  string |  |
| `database`  string |  |
| `org_name`  string |  |
| `column_length`  uint32 | column\_length is really a uint32. All 32 bits can be used. |
| `charset`  uint32 | charset is actually a uint16. Only the lower 16 bits are used. |
| `decimals`  uint32 | decimals is actualy a uint8. Only the lower 8 bits are used. |
| `flags`  uint32 | flags is actually a uint16. Only the lower 16 bits are used. |

### query.QueryResult

QueryResult is returned by Execute and ExecuteStream. As returned by Execute, len(fields) is always equal to len(row) (for each row in rows). As returned by StreamExecute, the first QueryResult has the fields set, and subsequent QueryResult have rows set. And as Execute, len(QueryResult[0].fields) is always equal to len(row) (for each row in rows for each QueryResult in QueryResult[1:]).

#### Properties

| Name | Description |
| --- | --- |
| `fields`  list <Field> | Field describes a single column returned by a query |
| `rows_affected`  uint64 |  |
| `insert_id`  uint64 |  |
| `rows`  list <Row> | Row is a database row. |
| `extras`  ResultExtras | ResultExtras contains optional out-of-band information. Usually the extras are requested by adding ExecuteOptions flags. |

### query.ResultExtras

ResultExtras contains optional out-of-band information. Usually the extras are requested by adding ExecuteOptions flags.

#### Properties

| Name | Description |
| --- | --- |
| `event_token`  EventToken | EventToken is a structure that describes a point in time in a replication stream on one shard. The most recent known replication position can be retrieved from vttablet when executing a query. It is also sent with the replication streams from the binlog service. |
| `fresher`  bool | If set, it means the data returned with this result is fresher than the compare\_token passed in the ExecuteOptions. |

### query.ResultWithError

ResultWithError represents a query response in the form of result or error but not both.

#### Properties

| Name | Description |
| --- | --- |
| `error`  vtrpc.RPCError | RPCError is an application-level error structure returned by VtTablet (and passed along by VtGate if appropriate). We use this so the clients don't have to parse the error messages, but instead can depend on the value of the code. |
| `result`  query.QueryResult | QueryResult is returned by Execute and ExecuteStream. As returned by Execute, len(fields) is always equal to len(row) (for each row in rows). As returned by StreamExecute, the first QueryResult has the fields set, and subsequent QueryResult have rows set. And as Execute, len(QueryResult[0].fields) is always equal to len(row) (for each row in rows for each QueryResult in QueryResult[1:]). |

### query.Row

Row is a database row.

#### Properties

| Name | Description |
| --- | --- |
| `lengths`  list <sint64> | lengths contains the length of each value in values. A length of -1 means that the field is NULL. While reading values, you have to accummulate the length to know the offset where the next value begins in values. |
| `values`  bytes | values contains a concatenation of all values in the row. |

### query.StreamEvent

StreamEvent describes a set of transformations that happened as a single transactional unit on a server. It is streamed back by the Update Stream calls.

#### Properties

| Name | Description |
| --- | --- |
| `statements`  list <Statement> | The statements in this transaction. |
| `event_token`  EventToken | EventToken is a structure that describes a point in time in a replication stream on one shard. The most recent known replication position can be retrieved from vttablet when executing a query. It is also sent with the replication streams from the binlog service. |

#### Messages

##### StreamEvent.Statement

One individual Statement in a transaction.

*Properties*

| Name | Description |
| --- | --- |
| `category`  Category |  |
| `table_name`  string | table\_name, primary\_key\_fields and primary\_key\_values are set for DML. |
| `primary_key_fields`  list <Field> | Field describes a single column returned by a query |
| `primary_key_values`  list <Row> | Row is a database row. |
| `sql`  bytes | sql is set for all queries. FIXME(alainjobart) we may not need it for DMLs. |

#### Enums

##### StreamEvent.Statement.Category

One individual Statement in a transaction. The category of one statement.

| Name | Value | Description |
| --- | --- | --- |
| `Error` | `0` |  |
| `DML` | `1` |  |
| `DDL` | `2` |  |

### query.Target

Target describes what the client expects the tablet is. If the tablet does not match, an error is returned.

#### Properties

| Name | Description |
| --- | --- |
| `keyspace`  string |  |
| `shard`  string |  |
| `tablet_type`  topodata.TabletType | TabletType represents the type of a given tablet. |

### query.Value

Value represents a typed value.

#### Properties

| Name | Description |
| --- | --- |
| `type`  Type |  |
| `value`  bytes |  |

### topodata.KeyRange

KeyRange describes a range of sharding keys, when range-based sharding is used.

#### Properties

| Name | Description |
| --- | --- |
| `start`  bytes |  |
| `end`  bytes |  |

### topodata.ShardReference

ShardReference is used as a pointer from a SrvKeyspace to a Shard

#### Properties

| Name | Description |
| --- | --- |
| `name`  string | Copied from Shard. |
| `key_range`  KeyRange | KeyRange describes a range of sharding keys, when range-based sharding is used. |

### topodata.SrvKeyspace

SrvKeyspace is a rollup node for the keyspace itself.

#### Properties

| Name | Description |
| --- | --- |
| `partitions`  list <KeyspacePartition> | The partitions this keyspace is serving, per tablet type. |
| `sharding_column_name`  string | copied from Keyspace |
| `sharding_column_type`  KeyspaceIdType |  |
| `served_from`  list <ServedFrom> |  |

#### Messages

##### SrvKeyspace.KeyspacePartition

*Properties*

| Name | Description |
| --- | --- |
| `served_type`  TabletType | The type this partition applies to. |
| `shard_references`  list <ShardReference> | ShardReference is used as a pointer from a SrvKeyspace to a Shard |

##### SrvKeyspace.ServedFrom

ServedFrom indicates a relationship between a TabletType and the keyspace name that's serving it.

*Properties*

| Name | Description |
| --- | --- |
| `tablet_type`  TabletType | ServedFrom indicates a relationship between a TabletType and the keyspace name that's serving it. the tablet type |
| `keyspace`  string | the keyspace name that's serving it |

### vtrpc.CallerID

CallerID is passed along RPCs to identify the originating client for a request. It is not meant to be secure, but only informational. The client can put whatever info they want in these fields, and they will be trusted by the servers. The fields will just be used for logging purposes, and to easily find a client. VtGate propagates it to VtTablet, and VtTablet may use this information for monitoring purposes, to display on dashboards, or for blacklisting purposes.

#### Properties

| Name | Description |
| --- | --- |
| `principal`  string | principal is the effective user identifier. It is usually filled in with whoever made the request to the appserver, if the request came from an automated job or another system component. If the request comes directly from the Internet, or if the Vitess client takes action on its own accord, it is okay for this field to be absent. |
| `component`  string | component describes the running process of the effective caller. It can for instance be the hostname:port of the servlet initiating the database call, or the container engine ID used by the servlet. |
| `subcomponent`  string | subcomponent describes a component inisde the immediate caller which is responsible for generating is request. Suggested values are a servlet name or an API endpoint name. |

### vtrpc.RPCError

RPCError is an application-level error structure returned by VtTablet (and passed along by VtGate if appropriate). We use this so the clients don't have to parse the error messages, but instead can depend on the value of the code.

#### Properties

| Name | Description |
| --- | --- |
| `code`  ErrorCode |  |
| `message`  string |  |

Contact: vitess@googlegroups.com  **·**  
Announcements  **·**  
© 2017 Vitess powered by Google Inc
